# Association of Ambient Temperature and Absolute Humidity with the Effective Reproduction Number of COVID-19 in Japan

**DOI:** 10.3390/pathogens12111307

**Published:** 2023-11-01

**Authors:** Keita Wagatsuma

**Affiliations:** 1 Division of International Health (Public Health), Graduate School of Medical and Dental Sciences, Niigata University, Niigata 951-8510, Japan; waga@med.niigata-u.ac.jp; Tel.: +81-25-227-2129; 2 Japan Society for the Promotion of Science, Tokyo 102-0083, Japan

**Keywords:** SARS-CoV-2, transmissibility, ambient temperature, absolute humidity, epidemics

## Abstract

This study aimed to quantify the exposure-lag-response relationship between short-term changes in ambient temperature and absolute humidity and the transmission dynamics of severe acute respiratory syndrome coronavirus 2 (SARS-CoV-2) in Japan. The prefecture-specific daily time-series of newly confirmed cases, meteorological variables, retail and recreation mobility, and Government Stringency Index were collected for all 47 prefectures of Japan for the study period from 15 February 2020 to 15 October 2022. Generalized conditional Gamma regression models were formulated with distributed lag nonlinear models by adopting the case-time-series design to assess the independent and interactive effects of ambient temperature and absolute humidity on the relative risk (RR) of the time-varying effective reproductive number (*R_t_*). With reference to 17.8 °C, the corresponding cumulative RRs (95% confidence interval) at a mean ambient temperatures of 5.1 °C and 27.9 °C were 1.027 (1.016–1.038) and 0.982 (0.974–0.989), respectively, whereas those at an absolute humidity of 4.2 m/g^3^ and 20.6 m/g^3^ were 1.026 (1.017–1.036) and 0.995 (0.985–1.006), respectively, with reference to 10.6 m/g^3^. Both extremely hot and humid conditions synergistically and slightly reduced the *R_t_*. Our findings provide a better understanding of how meteorological drivers shape the complex heterogeneous dynamics of SARS-CoV-2 in Japan.

## 1. Introduction

Since the first detection of the severe acute respiratory syndrome coronavirus 2 (SARS-CoV-2) in Wuhan, China at the end of 2019, the pandemic of the coronavirus disease 2019 (COVID-19) has continued for more than three years, leading to a devasting loss of life [1,2]. With the rapid increase in COVID-19 incidence, the Japanese government, on 16 April 2020, declared a state of emergency, which imposed a voluntary reduction in physical contact that effectively helped to control the epidemic in 2020 [3,4]. However, when socioeconomic activities resumed in late May 2020, COVID-19 cases began to re-emerge. Despite a temporary decline in morbidity, a third wave of cases started from late October 2020 [5]. In response, from late November 2020 onward, prefectures that had high numbers of COVID-19 patients asked bars and restaurants to reduce their hours of operation [6]. However, infection spread continued, and the Japanese government declared a second state of emergency on 7 January 2021 wherein the general public was instructed to refrain from non-essential outings [7]. The extended period of the pandemic was fueled by the emergence of variants of concern, including the Alpha (B.1.1.7), Delta (B.1.617), and Omicron (B.1.1.529) variants, which were prevalent in Japan from March to May 2021, June to December 2021, and January 2022 onward, respectively; they had higher transmissibility and greater intrinsic severity than the wild type [8]. Overall, the epidemiology of SARS-CoV-2 in Japan had a total of seven waves by 2022, with each wave ultimately controlled by public health and social measures and vaccination, but new waves were repeatedly triggered by the resumption of social activities and the appearance of novel variants [9]. 

Climate variability remains a critical global challenge that may alter ambient temperature and precipitation patterns, increase the risk of extreme meteorological conditions, and, in the long term, change local hydrological conditions. To date, epidemiological studies have demonstrated a complex exposure-response relationship between meteorological drivers and SARS-CoV-2 transmission dynamics; however, the relative importance of these driver remains debatable and other details of the association remain unexplored [10,11,12,13,14,15]. Despite the conflicting results of several epidemiological studies, some clear trends on the role of ambient temperature and humidity (absolute and relative) have emerged. Furthermore, many global modeling analyses have supported local findings on a negative association between ambient temperature and relative humidity as meteorological drivers and COVID-19 incidence in 20, 166, and 127 countries as well as 1236 regions globally from data collected until April, March, August, and May 2020, respectively [10,16,17,18,19]. In addition, a subsequent development study, which evaluated 188 countries until December 2020, showed similar negative exposure-response associations as those identified in the previous studies, and further highlighted the importance of meteorological drivers in SARS-CoV-2 transmission dynamics [11]. Some of the studies that have investigated these exposure-response relationships had methodological weaknesses and generated conflicting results [20,21]. In Japan, a study conducted in Tokyo reported a cumulative relative risk (RR) of 1.3 at the first mean ambient temperature percentile (i.e., 3.3 °C) [22]. For instance, the conflicting findings include positive [23,24], negative [25,26], nonlinear [27,28], and non-significant [29,30] associations. Other studies suggested N-shaped [12] or U-shaped [11] associations between relative humidity and COVID-19 incidence. Nonetheless, owing to the relatively narrow range and fluctuation of meteorological drivers in different observation periods, these results should be interpreted cautiously. Furthermore, effect modification (i.e., interactive effect) among meteorological drivers should be considered; however, there is limited epidemiological evidence [31].

The variability in the results of previous studies may be partly explained by differences in the sample size, observation period, breadth of the spatiotemporal scale of analysis, the application of different statistical modeling techniques with varying degrees of sophistication, and the degree of consideration of potential confounding factors [16,32]. In particularl, previous systematic reviews have indicated that these modeling studies have significant methodological limitations that may introduce bias and limit causal inferences [33,34]. Many studies did not evaluate the possibility of a nonlinear association and temporally lagged effects of the drivers and outcomes, did not account for time-varying confounders, or did not consider location-specific confounders. Generally, the effects of meteorological drivers and disease transmission are complex, nonlinear, and often delayed by several days to weeks, which limits the statistical inferences that can be obtained from traditional linear modeling methods [35,36]. In particular, the time-varying distributed lag nonlinear models (DLNM), which were developed in the last decade, have been relatively underused despite being well suited for time-series statistical modeling of infectious disease dynamics [37,38]. Moreover, despite the advantages of the method, DLNM has not been overly used in research, and only some disease modeling studies have utilized it [16,32,39,40,41]. 

Although epidemiological studies have investigated associations between climate variability and risk of transmission, the association between hydro-meteorological hazards and outbreaks of diseases, and the delayed effects of such conditions on transmission, are poorly understood. To fill this knowledge gap, this study aimed to model the complex non-linear and temporally multi-delayed relationships between multiple meteorological drivers (such as ambient temperature and absolute humidity) and the time-varying effective reproduction number (*R_t_*), defined as the expected number of secondary cases arising from a single primary case at calendar time *t* during the multiple epidemiological years of the pandemic across a total of all 47 Japanese prefectures, analyzing the readily accessible empirical data and adopting flexible time-series statistical modelling approaches. Furthermore, the modification role of absolute humidity in the abovementioned association was ascertained. These findings provide an epidemiological profile of the transmission dynamics to enable public health policy decision-making and strategies to impede SARS-CoV-2 transmission.

## 2. Materials and Methods

### 2.1. Design Setting

This time-series statistical modeling study investigated the possible nonlinear and multi-delayed associations between the time-varying transmissibility of SARS-CoV-2 and environmental drivers (i.e., ambient temperature and absolute humidity) during approximately 2.5 epidemiological years (from 15 February 2020 to 15 October 2022) of the pandemic in Japan in the context of an ecological study. Japan is located on latitudes and longitudes from approximately 26° N to 43° N and 127° E to 141° E, respectively, in the Western Pacific Region, and comprises a total of 47 prefectures (Figure S1).

### 2.2. Empirical Datasets

#### 2.2.1. Epidemiological Data

In the present study, prefecture-specific daily time-series data of newly confirmed and notified COVID-19 cases for all 47 Japanese prefectures during the study period (from 15 February 2020 to 15 October 2022) were retrieved. These locations and periods were selected based on the optimum period, while simultaneously considering the geographical diversity in Japan and the ability to account for multiple crucial confounders (e.g., meteorological drivers, mobility, and restrictions). The dates of the reports were systematically collected from the official COVID-19 case reports that were made available online by the Ministry of Health, Labor and Welfare, Japan (MHLW) [42]; these data were supplemented and verified with information from the press releases of other reporting agencies (e.g., prefectural office). In Japan, according to The Infectious Disease Control Law, COVID-19 has been designated as a notifiable disease; therefore, it is mandatory to report every laboratory-confirmed notified COVID-19 case by validated testing methods, including reverse transcriptase polymerase chain reaction (RT-PCR) or rapid diagnostic testing of nose or throat swabs. Newly detected COVID-19 cases are notified to the public health centers, the epidemiological and clinical information are retrieved from each recorded notification, and the press release information is published on the official website based on the notification system [43].

#### 2.2.2. Meteorological Data

In this study, prefecture-specific daily time-series meteorological data of all 47 Japanese prefectures were retrieved from the Automated Meteorological Data Acquisition System (AMeDAS) for the study period [44]. Daily meteorological data including mean ambient temperature (°C), relative humidity (%), precipitation (mm), and wind speed (m/s) published on the website were utilized. Using the mean ambient temperature and relative humidity, the daily mean absolute humidity (m/g^3^) was calculated [45]. To model the impact of accurate humidity, absolute humidity was evaluated because it is independent of ambient temperature. Daily meteorological data collected from meteorological observatories situated in the prefectural capital city were used for each prefecture.

#### 2.2.3. Mobility Data

In this study, prefecture-specific daily time-series of Google’s COVID-19 Community Mobility Reports data during the study period were retrieved [46]. For Google’s mobility data, the measure of mobility is reported for six data streams: grocery and pharmacy mobility, parks mobility, residential mobility, retail and recreation mobility, transit station mobility, and workplace mobility. As these types of mobility data may not accurately describe associations with SARS-CoV-2 transmission dynamics, mobility patterns associated with retail and recreation were utilized in the present analyses based on previous empirical domain knowledge that this category likely represents mobility in close contact settings that are associated with SARS-CoV-2 transmission dynamics [3,47,48]. This measure quantifies the percentage deviation (%) from the median baseline for each day of the week for 5 weeks (3 January 2020 to 6 February 2020).

#### 2.2.4. Restrictions

To control the time-varying intensity of public health and social measures, daily data on the GSI were retrieved from the OxCGRT project at the national level (scale: 1–100, with higher values representing stringent measures implemented to hinder transmission) [49]. These data represent an overall GSI based on public health and social measures (e.g., school closing, workplace closing, canceling of public events, restrictions on gathering sizes, closing public transport, stay-at-home requirements, restrictions on internal movement, and international travel controls) and other restriction indicators. 

### 2.3. Statistical Analysis

#### 2.3.1. Descriptive Descriptions

The trends of time-series variations in the number of new daily COVID-19 cases, meteorological variables (i.e., mean ambient temperature, absolute humidity, precipitation, and wind speed), mobility pattern (i.e., retail and recreation mobility patterns), and the GSI from the OxCGRT during the study period were visually assessed to determine the key characteristics of the dataset included in this study. The probability distributions of the time-series data for these dependent and independent variables were described by utilizing the following descriptive statistics: mean, SD, minimum (Min), 25th percentile (P_25_), 50th percentile (P_50_), 75th percentile (P_75_), and maximum (Max).

#### 2.3.2. Estimation of Time-Varying Transmissibility 

To assess the time-varying transmissibility of SARS-CoV-2 throughout an epidemic, the time-varying *R_t_* was estimated from the incident time-series surveillance data by applying the simple branching process model in the context of Bayesian inference [50]. *R_t_* can be estimated by the ratio of the number of new infections generated at time step *t* (*I_t_*) to the total infectiousness of the infected individuals at time *t*, given by $\sum_{s=1}^{t}I_{t-a}w_{s}$, where the sum of infection incidence up to the time step *t* − 1 is weighted by the infectivity function *w_s_*. *R_t_* is the average number of secondary cases that each infected individual would infect if the conditions remained as they were at time *t* and is thus commonly used to characterize pathogen transmissibility during an epidemic. A value of *R_t_* greater than one typically signifies an increasing number of cases and the ongoing spread of the infection, whereas an *R_t_* of less than one indicates a decline in the spread of the infection. The *R_t_* value can be adjusted to include the serial interval (SI), which is the gap between the onset of the index and the next case, as an infectivity function while assuming a gamma distribution [50,51]. Here, we utilized an SI with a mean of 4.7 days and a SD of 2.9 days, as documented by Nishiura et al. [52]. The selection of the smoothing window is determined by a trade-off between temporal resolution and credible interval width around the resulting estimates; herein, the time-varying estimates were made with a 7-day sliding window. Statistical estimation was performed using R statistical programming software version 4.1.0 with the “EpiEstim” package.

#### 2.3.3. Construction of the Time-Series Statistical Model 

The time-series statistical model was built in multiple stages in order to build a robust, reliable model. Prior to constructing the model, the probability distribution of the dependent variable—the daily time-varying *R_t_* (the normality of probability distribution was assessed by the Shapiro–Wilk test)—was ascertained (Figure S2) and the relationships (e.g., linearity) between daily time-varying *R_t_* and each independent variable were assessed. All dependent variables and independent variables included in the statistical models were assessed for multicollinearity by using the pairwise Spearman’s rank-order cross-correlation coefficient (*ρ*). If the variables were highly linearly correlated (cutoff: |*ρ*| > 0.8), the variable with the largest mean absolute statistical correlation with the other independent variables was removed [53]. In the preliminary analysis, a statistically strong linear correlation was observed between the mean ambient temperature and absolute humidity (*ρ* = 0.95); thus, separate independent models were constructed (Table S1).

In this present study, a multivariate generalized conditional regression model with a Gamma distribution and logarithmic-link function, allowing for overdispersion in the observational epidemiologic data, combined with time-varying DLNM, was specified to implement the cutting-edge CTS analysis. A stratum was designed by a three-way interaction term of the year, calendar month, and prefecture to compare exposure levels between case and control days that were matched within the stratum. Thus, the CTS design combined a longitudinal structure while controlling for time-varying and time-invariant confounders between the study sites [54]. This method was used to discern the underlying association between the time-varying transmissibility (i.e., *R_t_*) and different environmental drivers (i.e., mean ambient temperature and absolute humidity) as main exposures in a primary model. The class of DLNM models introduces a cross-basis function, which can be obtained by calculating the tensor product of basis functions and describes the dependent variable’s probability distribution in the independent variable dimension and the lagging dimension to simultaneously assess the lag and nonlinear effects of the time-varying exposure driver [38].

The association of the time-varying *R_t_* with different meteorological drivers (i.e., mean ambient temperature and absolute humidity) was formulated by designing a cross-basis function of DLNM, which was obtained by the combination of two functions for modeling the potentially nonlinear exposure-response and the additional lag-response associations, respectively, that examine multi-delayed effects [38]. More specifically, exposure-response associations were modeled using a natural cubic spline with 3 degrees of freedom (df); the lag-response association was modeled using a natural cubic spline with 3 df to allow enough flexibility to capture potentially complex associations between meteorological indicators and their multi-delayed impact on risk. Based on the previously reported incubation period (5–12 days) [55,56] and potential reporting delay, temporal lags (i.e., delays in potential effect) of up to 14 days were considered as the default lag for the cross-basis function of each independent variable (i.e., mean ambient temperature and absolute humidity) related to the dependent variable. 

Multiple confounders were considered in the main model. First, a natural cubic spline function with 3 df for precipitation (continuous) and wind speed (continuous) was formulated to adjust for the nonlinear confounding effects of the other meteorological variables. Additionally, retail and recreation mobility were considered as possible confounders because of their association with the time-varying *R_t_* (via population mixing) as well as the exposures (e.g., weather-dependent free time outdoor mobility), and they are not on the causal pathway between the exposures and the outcome. The mobility term was modeled by distributed lag linear models (DLMs) with a lag of 0–14 days, where the lag dimension was modeled with a natural cubic spline with one internal knot. Changes in the national-level government public health and social measures were controlled by a linear term that incorporated the GSI from the OxCGRT. Furthermore, varying baseline risk on top of shared long-term seasonal variation and cycle as well as short-term trends were modeled by incorporating natural cubic splines of time (6 df per year for the main analysis), week of year (category), day of week (category), and public holiday (category) fixed effects variables as possible confounders [16,32,39,57]. The autocorrelation term of residuals in the case of infectious disease is pathogen-specific and needs to be accounted for; therefore, autoregressive terms at the logarithm of the order of one to five were incorporated into the statistical models [36]. 

#### 2.3.4. Sensitivity Analysis 

To assess the robustness of the study’s findings, multiple sensitivity analyses were performed. First, the analysis was repeated by varying the natural cubic spline of time from 6 to 4 df per year. Next, a sensitivity analysis of the observed effect on the days of lags accounted were performed by varying the length of the lag period from 14 to 7 days. Additionally, the functional form of spline for the exposure-response curve was altered from a natural cubic spline with 3 df to a second-degree polynomial. The lag–response curve was remodeled with a natural cubic spline with intercept and two equally spaced knots on a log scale. Finally, the functional form of spline was changed from a natural cubic spline with 3 df to a quadratic B-spline with three internal knots that were placed at the 25th, 50th, and 75th percentiles of mean ambient temperature and absolute humidity. For all statistical models, estimates were quantified as cumulative-day (i.e., 0–14 days), and single-day (i.e., 0, 7, and 14 days) RRs (95% CI at the 10th and 90th percentiles) of the mean ambient temperature, and absolute humidity values were used to present the strengths of the associations. To assess the modification role of absolute humidity in the mean ambient temperature *R_t_* nonlinear association, a linear interaction between the cross-basis function of ambient temperature and the different absolute humidity group (high, medium, and low) was additionally entered into the final model. To understand variations in the risk for different scenarios, the absolute humidity in the interaction term was centered on the 10th (low levels), 50th (intermediate levels), and 90th (high levels) percentile of the value range in 47 Japanese prefectures to extract the effect-size estimates of mean ambient temperature (corresponding to the 5th, 10th, 90th, and 95th percentiles) for time-varying *R_t_*. The likelihood ratio tests (accounting for overdispersion) assessed differences in risk between groups. Using the observed temporal mean trends of time-varying *R_t_* across all 47 Japanese prefectures with the predicted values from the main model, the goodness of fit in intra-interval predictions of the model was examined. Owing to the associations being generally nonlinear and multi-delayed, the overall median values (50th percentile) of mean ambient temperature and absolute humidity were utilized as the reference levels. Statistical significance was considered at *p* < 0.05 (i.e., type I error) on a two-tailed test. All analyses were performed in the R statistical programming software version 4.1.0 (R Foundation for Statistical Computing, Vienna, Austria) using “dlnm” for the DLNM framework and “gnm” for the generalized conditional regression model. 

### 2.4. Ethical Considerations 

The present study analyzed publicly available data, and the datasets used in this study were de-identified and fully anonymized in advance. The analysis of publicly available data without patient-identifying information did not require ethical approval. The present study was conducted in accordance with the principles of the Declaration of Helsinki (as revised in 2013).

## 3. Results

### 3.1. Descriptive Statistics

The dataset included 21,472,376 cases that were notified from 47 Japanese prefectures during the study period from 15 February 2020 to 15 October 2022 (Table 1). The mean number of the daily newly confirmed cases was 469 (range: 0–40,406) per prefecture, and the mean daily *R_t_* averaged over all prefectures and days during the study period was 1.24 (0.04–78.04). As expected, the mean daily ambient temperature, absolute humidity, precipitation, wind speed, retail and recreation mobility, and GSI from the OxCGRT varied widely between the prefectures. Specifically, the observed range of values for these variables was as follows: mean ambient temperature (−10.40 to 33.00 °C), absolute humidity (11.67–26.22 m/g^3^), precipitation (0.00–306.00 mm), windspeed (0.50–17.90 m/s), retail and recreation mobility (−89.00 to 66.00%), and GSI from the OxCGRT (19.44–55.09%). Overall, the mean ambient temperature and absolute humidity showed a positive trend from spring to summer with a peak in August; clear seasonal patterns were observed; however, seasonal trends of precipitation, windspeed, retail and recreation mobilities, and the GSI from the OxCGRT showed less obvious trends (Figure S3). The pairwise Spearman’s rank-order linear correlation coefficients statistics showed that time-varying *R_t_* were weakly and negatively correlated with mean ambient temperature (*ρ* = −0.08) and absolute humidity (*ρ* = −0.06) across all prefectures in Japan (Table S2).

### 3.2. Exposure–Response Relationships between Meteorological Drivers and Transmissibility

**Figure 1:**
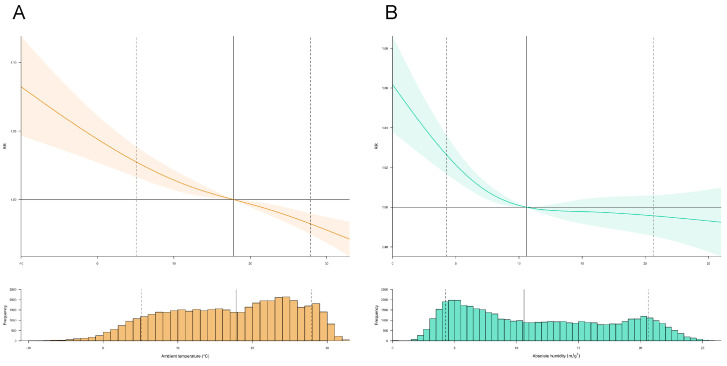
Overall exposure-response relationships among ambient temperature, absolute humidity, and time-varying transmissibility of SARS-CoV-2. Overall exposure-response curves for the associations of the 14-day cumulative relative risks (RR) of the estimated time-varying effective reproduction number (*R_t_*) of SARS-CoV-2 with (**A**) the mean ambient temperature (°C) relative to the overall median of the mean ambient temperature (i.e., 17.8 °C), with related distributions, and (**B**) absolute humidity (units: m/g^3^) relative to the overall median of mean ambient temperature (i.e., 10.6 m/g^3^), with related distributions. The solid gray lines are the overall median of the mean ambient temperature and absolute humidity, and the dashed gray lines are the 10th and 90th percentiles. Shaded areas on the curves are 95% confidence intervals (CI). The relevant selected estimates (RRs and its 95% CIs) are provided in Table 2.

Figure 1 displays the cumulative exposure-response curves that illustrate the relationship between mean ambient temperature, absolute humidity, and time-varying *R_t_* across Japan. The findings reveal an inverse linear association between ambient temperature and *R_t_*, indicating that lower mean ambient temperatures are nonlinearly linked to increased SARS-CoV-2 transmissibility (Figure 1A). The corresponding cumulative RR was 1.027 (95% CI: 1.016–1.038) at 5.1 °C (25th percentile), and 0.982 (95% CI: 0.974–0.989) at 27.9 °C (90th percentile) (Figure 1A, Table 2). Through two-dimensional contour plots, the risk of transmissibility of SARS-CoV-2 was found to be the highest within a short lead time (0–4 days) during extremely cold conditions (Figure S4A). Conversely, exceptionally high mean ambient temperatures were associated with a decreased RR of *R_t_* after a 14-day period. The analysis of lag-response relationships for different scenarios showed a decreasing trend in the lag dimension, with RRs attenuating to 0.997–1.001 around lag-day 14 (Figure S5A). An inverse association of low absolute humidity levels with an increased risk of SARS-CoV-2 transmissibility was observed (Figure 1B). Specifically, a nonlinear, inverted J-shaped association between absolute humidity and time-varying *R_t_* was observed when the humidity was lower than 10.6 m/g^3^, whereas at values above this level, the humidity was almost unrelated to transmissibility. The cumulative RRs were 1.026 (95% CI: 1.017–1.036) at an absolute humidity of 4.2 m/g^3^, and 0.995 (95% CI: 0.985–1.006) at 20.6 m/g^3^ with reference to 10.6 m/g^3^ (Figure 1B, Table 2). The increased RR of transmissibility was observed over a wide lag of 0–14 days with absolute humidity below 10.6 m/g^3^ (Figure S4B). Although heterogeneous associations exist above 10.0 m/g^3^, the protective effect of higher absolute humidity occurred at much shorter lags (0–3 days). The lag-response associations for scenarios with an absolute humidity of 4.2 m/g^3^ exhibited an inverse U-shaped trend in the lag dimension, reaching a peak RR of 1.002, with the smallest effects observed on lag days 0 and 14 (Figure S5B). The lag-response associations for scenarios with an absolute humidity of 4.2 m/g^3^ and scenarios with an absolute humidity of 20.6 m/g^3^ showed that the lag-response associations were almost constant.

### 3.3. Effect Modification of Absolute Humidity on the Exposure–Response Relationship between Mean Ambient Temperature and Time-Varying Transmissibility

**Figure 2:**
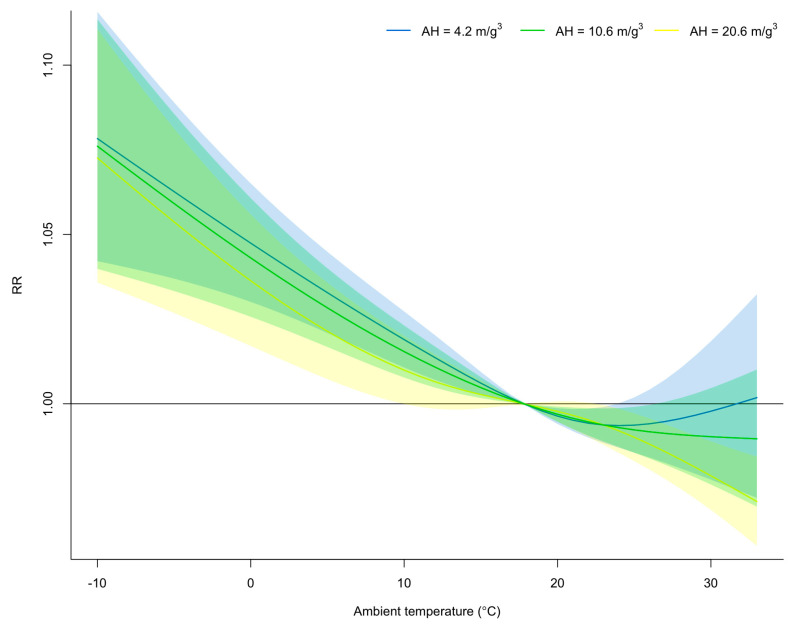
Overall exposure-response relationship between mean ambient temperature and time-varying transmissibility of SARS-CoV-2 by different level of absolute humidity. The pooled relative risk (RR) was centered at the overall median of mean ambient temperature (17.8 °C; 50th percentile); high-absolute-humidity (AH) group (20.6 m/g^3^; 90th percentile), medium (10.6 m/g^3^; 50th percentile), and low-AH group (4.2 m/g^3^; 10th percentile). Shaded areas on the curves indicate the 95% confidence intervals.

The present study examined the exposure-response relationship between the mean ambient temperature and time-varying *R_t_* across different levels of absolute humidity: high-absolute-humidity group (20.6 m/g^3^; 90th percentile), medium group (10.6 m/g^3^; 50th percentile), and low-absolute-humidity group (4.2 m/g^3^; 10th percentile). Generally, these results demonstrated that higher levels of absolute humidity were associated with lower values of time-varying *R_t_* (Figure 2). The figure indicates a difference in the risk between groups with high and low absolute humidity (*p* < 0.001, likelihood ratio test). Notably, the effect of the high-absolute-humidity group on time-varying *R_t_* was more pronounced when the mean ambient temperature exceeded 24.0 °C. For instance, at the 90th percentile of mean ambient temperature (i.e., 27.9 °C), the higher-absolute-humidity group had an approximately 1.2% lower risk of time-varying *R_t_* than the lower absolute humidity group (RR: 0.995 (95% CI: 0.981–1.010) versus RR: 0.983 (95% CI: 0.975–0.992); Figure 2, Table S2).

### 3.4. Further Investigations

The main findings described above were confirmed by repeating the series of sensitivity analyses to verify the robustness of the results. First, decreasing the df in the natural spline for long-term time trends from 6 to 4 df per year leads to more evident exposure-response associations (Figures S6 and S10). Also, changing the incorporation of 0–14 lag days down to 0–7 lag days revealed that the observed risk effect shape was substantially robust over the different parameterizations (Figures S7 and S11). The pooled effect estimates for the exposure-response association were robust, altering the model specifications (the different number of knots and the other functional form of the spline; Figures S8, S9, S12, and S13). Indeed, the time-series of predicted values from main model against the observed temporal mean trends of time-varying *R_t_* across all 47 Japanese prefectures exhibited the good fit of the model (*ρ* = 0.99 for both meteorological drivers; Figure S14).

## 4. Discussion

Despite COVID-19′s profound impact on global public health, little is known about how the virus transmits from person to person and what environmental and meteorological conditions make this process more likely. In this study, a cutting-edge CTS design was utilized by embedding a DLNM to systematically explore the nonlinear and delayed associations between multiple meteorological drivers (i.e., mean ambient temperature and absolute humidity) and the time-varying *R_t_* of SARS-CoV-2 across all 47 Japanese prefectures and to further investigate the modification role of absolute humidity on the associations. This nationwide study presents one of the most thorough assessments of the influence of meteorological drivers on the seasonality of SARS-CoV-2 across a large gradient of meteorological conditions related to latitudes and longitudes. Evidence of a modest negative non-monotonic association of mean ambient temperature and absolute humidity with *R_t_* over lags of 0–14 days was obtained. With time-series statistical modeling, both higher mean ambient temperature and higher absolute humidity were found to be nonlinearly associated with decreased *R_t_*. Furthermore, evidence of the effect modification suggested that the influence of mean ambient temperature on *R_t_* increases at higher absolute humidity levels. Moreover, the results highlight the relative role played by environmental drivers and indicate an additional mechanism that is possibly involved in shaping time-varying transmission heterogeneity and is yet to be identified. 

Of note, the findings on the exposure-response relationship between mean ambient temperature, absolute humidity, and transmission dynamics of SARS-CoV-2 are largely consistent with those of the previous series of modelling studies. A systematic review of 61 studies conducted at the end of 2020, most studies reporting a linear trend, documented a negative association between the incidence of COVID-19 and ambient temperature (33 versus 6 studies) and humidity (13 versus 3 studies) [58]. Another systematic review of approximately 160 other studies found an almost even split of studies that discovered a positive or negative relationship between ambient temperature and transmission [59]. Indeed, multiple global analyses from the beginning of the pandemic to the present support these local findings, including the current findings of this study on mean ambient temperature and humidity. A large time-series modeling study using DLNM that assessed 1,908,197 confirmed COVID-19 cases from 190 countries, including four major climate zones as of 13 April 2020, also observed an overall inverse association between mean ambient temperature (highest and lowest RRs at 5.0 °C and 20.0 °C, respectively) and incidence, supporting these present findings [12]. However, a different exposure-risk association was observed for relative humidity (RR peak at 72.0%). More notably, a recently latest global modelling study from the Multi-City Multi-Country Network involved a detailed city-level analysis of meteorological drivers and incidence at 455 cities across 20 countries, suggesting that comparatively low ambient temperatures and low absolute humidity were significantly associated with increased risks of incidence, in contrast to relative humidity [16]. This study underscores the regional heterogeneity of weather-related effects on transmission. Additionally, Fontal et al. analyzed the transitory associations of mean ambient temperature and absolute humidity until October 2020 in 10 world regions and obtained negative associations for both [60]. Meanwhile, in addition to these existing studies, there are some time-series modelling studies that use time-varying transmissibility (*R_t_*) to quantify more rigorously the association with environmental exposure utilizing DLNM. A crucial global city-level study by Sera et al., which analyzed the transmission in 409 cities in 26 countries across the globe during the early stages of the pandemic, identified a non-linear (although primarily downward) association between ambient temperature and absolute humidity with *R_t_*, and concluded that 2.4% and 2.0% of the variation in *R_t_* are attributable to these meteorological drivers, respectively [41]. Another two studies on United States (US) counties only also found elevated infection risks (increased *R_t_* values) at lower ambient temperatures, and one of them also for lower specific humidifies [28,40]. Overall, the findings of the present study complement those of the existing literature, which shows that a negative non-linear association is observed with higher ambient temperatures and higher absolute humidity, with lower incidence or *R_t_*. However, current modelling theory does not fully explain the non-linear behavior of the exposure-response relationship, especially why it varies from study to study. 

With regard to the non-linear effects of mean ambient temperature and humidity, evidence has also been accumulating on the mechanism from several possible biological and behavioral aspects. Several previous studies have confirmed the observation that lower ambient temperatures increase the transmission rate of viral diseases, and the biophysical theories and laboratory results suggest that low temperatures increase the stability and survival of virus particles. For instance, laboratory studies conducted by Chin et al. reveal the stability of SARS-CoV-2 at approximately 4.0 °C, whereas higher temperatures of 70.0 °C lead to virus inactivation within 5 min, supporting epidemiological findings [61]. Besides the host’s influence, the virus’s viability outside the host’s body is also anticipated to be adversely affected by virus inactivation resulting from the degradation of the lipid layer at elevated ambient temperatures [62,63]. Furthermore, studies using animal experiments suggest that low temperatures may affect the potential ability of the host immune system to confront respiratory viruses, as blood circulation is reduced, and adaptive immunity is locally compromised [64,65]. In contrast, the association between low humidity and high levels of infections can be explained by the short ballistic settling characteristics of virus-containing droplets under wet conditions, whereas in contrast, under dry conditions, droplets can evaporate to form dry nuclei and remain suspended for long periods of time [66]. These findings align with the results of prior investigations highlighting the predictive role of absolute humidity in influenza transmission [67,68,69]. However, due to the high correlation between mean ambient temperature and absolute humidity (*ρ* = 0.95) in this modelling study, it is difficult to separate the effects of the two exposures and it is possible that the association of one merely reflects confounding of the other. Altogether, the current findings align with existing accumulated evidence from epidemiological, ecological, and laboratory studies, indicating that elevated mean ambient temperatures and absolute humidity may have contributed to the diminished viability of the virus and partially suppressed its transmission in Japan; however, it is necessary to recognize that the state of scientific knowledge on this topic is constantly evolving, as the literature in this area is broad and growing.

To our knowledge, little evidence has been accumulated on the effects of potential interactions in meteorological drivers on the transmission dynamics of SARS-CoV-2, and only conflicting and limited findings are available. The findings of this study suggest that absolute humidity had an intrinsic modification role in in the association of mean ambient temperature with time-varying *R_t_* across Japan. For instance, a systematic review including 517 literatures about these association concluded that hot and wet climates have a protective effect, supporting the results [70]. Using the time-series data by 10 September 2021, a one study analyzing 31,662 confirmed COVID-19 cases in Sydney, Australia also reported that incidences were influenced by ambient temperature when the humidity was low, and that cases increased in dry and warm conditions, generally consistent with the results of the present study [71]. In contrast, the only global modelling study to examine the impact of potential interactions of meteorological drivers on 2143 city- and district-level facilities in six low- and middle-income countries in 2020 reported that high ambient temperature and high relative humidity interacted with each other and were associated with increased risk in temperate climates but decreased risk in tropical climates [72]. Indeed, the impact of meteorological drivers on the dynamics of SARS-CoV-2 may vary depending on climatic zone [73]. These differences in ambient temperature effects may be partly explained by heat adaptation in different climatic zones; indeed, heat adaptation is formed by constant exposure to heat in tropical climates, whereas people living in temperate climates remain vulnerable to extremes of heat [74,75]. Overall, further detailed studies are warranted to evaluate the interactive effects of meteorological drivers that were exposed.

The present study has some notable strengths. The analysis was based on a large representative dataset at the national level consisting of daily time-series values from all 47 prefectures across Japan for 974 daily periods. A large sample size increased the validity of this study’s findings and helped investigate the overall risk by considering the spatiotemporal heterogeneity of the gradient of meteorological conditions from north to south in Japan. Moreover, complex DLNM parametrization to explore the exposure-response relationships between meteorological drivers and transmission dynamics was considered. This approach enabled the simultaneous assessment of complex associations, including immediate, delayed, and cumulative effects, and reduced the spatiotemporal trends by using a time-stratified sample to define strata based on the month, calendar year, and prefecture in the time series. Furthermore, multiple ecological and time-varying confounders were incorporated, and the less commonly studied variables of precipitation and wind speed were considered in the model.

Despite providing evidence for the relative importance of meteorological drivers in shaping complex disease dynamics, this study has several limitations. First, it should be noted that the present study provided an epidemiological finding based on the analysis of secondary observational data and regarded as a type of ecological study in statistical causal inference. Second, the present study used reporting dates instead of actual infection dates or symptom onset dates for calculating time-varying *R_t_*, which may have introduced biases attributed to reporting delays. Third, assuming spatial homogeneity within a prefecture, weather variables measured from a single monitoring station for each prefecture were used as representative data. Fourth, this study’s limited scope, which examined specific Japanese prefectures during a limited timeframe, may have restricted the generalizability of its findings to different epidemic periods and regions. Fifth, the present study utilized specific mobility data streams from the Google’s COVID-19 Community Mobility Reports as a surrogate measure of actual human mobility pattern [46]. Indeed, we have previously carried out time-series modelling analyses over a two-year period for six prefectures in Japan and found that retail and recreation mobility, as well as mean ambient temperature, had a greater impact. Specifically, mean ambient temperature explained 11.6% (95% CI: 5.9–17.7%) of the variation in time-varying *R_t_* compared to 19.6% (95% CI: 12.6–27.1%) for retail and recreation mobility. Modelling the impact of human mobility was outside the scope of this study, as it focused on assessing key meteorological factors for SARS-CoV-2, including mean ambient temperature and absolute humidity, but the combined impact of these needs to be clarified in the future. Sixth, there may exist endogeneity issues caused by unmeasured potential confounders. Indeed, the socioeconomic and demographic factors such as age and sex may have modified the complex dynamics [76]. Additionally, other potential drivers such as the different specific nonpharmaceutical interventions (e.g., social distancing interventions, physical self-isolation, school closure, hand hygiene behavior, and voluntary mask utilize) adopted by prefectures at different stages, vaccination, and other environmental drivers (e.g., ultraviolet levels and air pollutants) were not explicitly considered in the statistical model [13,30,57,77,78]. Particularly, although we utilized precipitation, wind speed, retail and recreation mobility, and GSI from the OxCGRT as main confounders in the models, other environmental drivers such as particulate matter (≤2.5 μm in diameter (PM_2.5_) and ≤10 μm in diameter (PM_10_)) may have also significantly contributed to dynamics of SARS-CoV-2 [79]. This study selected these confounders owing to the difficulty of obtaining systematic time-series data on air pollutants across Japan; therefore, further studies are needed to quantify the contribution of these drivers to the transmission dynamics of SARS-CoV-2 and to understand the interrelationships. Finally, the present study utilized a fixed SI (i.e., mean (SD): 4.7 (2.9) days) estimated in the early stages of the pandemic to quantify the time-varying *R_t_* of SARS-CoV-2 [52]. One study described a mean SI of 3.5 and 3.0 days for the Omicron variants, a little shorter than the tentative estimates reported previously, indicating that the estimates for *R_t_* may vary slightly [80]. Further data-driven modeling by time-varying transmissibility of SARS-CoV-2 transmission dynamics is subject for our studies by extending the methods described above.

## 5. Conclusions

Although the present findings did not directly represent a causal connection, the present study clarified important empirical evidence of nonlinear and delayed associations of SARS-CoV-2 transmission dynamics with meteorological drivers (i.e., mean ambient temperature and absolute humidity) during the COVID-19 pandemic in Japan. Moreover, the study identified the modification role of absolute humidity on the mean ambient temperature-related infection transmissibility and suggested that both extremely hot and humid conditions may synergistically and slightly reduce the risk of transmission. Monitoring and forecasting the occurrence, intensity, and evolution of hydrometeorological hazards is crucial for public health agencies in their efforts to prepare, mitigate, and manage responses to SARS-CoV-2 transmission dynamics and this epidemiological evidence can also provide implications for the related public health decisions, such as the establishment of weather-based disease early warning system. 

## Figures and Tables

**Table 1:** Descriptive statistics for the number of daily newly confirmed COVID-19 cases, effective reproduction numbers, meteorological variables, and mobility patterns, and the OxCGRT across all 47 Japanese prefectures and days.

Potential Drivers	Mean	Min	P_25_	P_50_	P_75_	Max
Daily newly confirmed cases	469	0	1	18	239	40,406
Effective reproduction number	1.24	0.04	0.84	1.02	1.31	78.04
Mean ambient temperature (°C)	17.02	−10.40	10.20	17.80	24.20	33.00
Absolute humidity (g/m^3^)	11.67	0.00	6.10	10.60	17.10	26.22
Precipitation (mm)	5.11	0.00	0.00	0.00	2.50	306.00
Wind speed (m/s)	2.87	0.50	2.00	2.60	3.40	17.90
Retail and recreation mobility (%)	−17.44	−89.00	−13.00	−7.00	−2.00	66.00
GSI from the OxCGRT (%)	42.59	19.44	37.04	45.37	47.22	55.09

Abbreviations: Min: minimum; P_25_, 25th percentile; P_25_, 50th percentile; P_75_, 75th percentile; Max, maximum; GSI, Government Stringency Index; OxCGRT, Oxford Coronavirus Government Response Tracer.

**Table 2:** Forecasting the specific relative risks of non-linear associations of mean ambient temperature and absolute humidity with time-varying transmissibility of SARS-CoV-2 in Japan.

Potential Drivers	Lag (Days)			
	0	7	14	0−14
	RR (95% CI)	RR (95% CI)	RR (95% CI)	RR (95% CI)
Mena ambient temperature (°C)				
5.1 °C	1.009 (1.004−1.015)	1.004 (1.001−1.007)	1.002 (0.996−1.008)	1.027 (1.016−1.038)
27.9 °C	1.008 (1.003−1.013)	1.004 (1.001−1.007)	1.002 (0.997−1.007)	0.982 (0.974−0.989)
Absolute humidity (m/g^3^)				
4.2 m/g^3^	1.007(1.000−1.007)	1.006 (1.002−1.006)	1.006 (0.999−1.006)	1.026 (1.017−1.036)
20.6 m/g^3^	1.002 (1.000−1.005)	1.003 (1.001−1.004)	1.001 (0.999−1.004)	0.995 (0.985−1.006)

Abbreviations: RR, relative risk; CI, confidence interval. Notes: 5.1 °C and 27.9 °C correspond to the 10th and 90th percentiles of mean ambient temperature, respectively; 4.2 and 20.6 m/g^3^ correspond to the 10th and 90th percentiles of absolute humidity, respectively. Associations between the estimated effective reproductive number (*R_t_*) and mean ambient temperature (°C) and absolute humidity (m/g^3^) are described as RR (95% CI) with reference to 17.8 °C and 10.6 m/g^3^ respectively.

## Data Availability

An anonymized dataset that enables replication of the analysis is publicly accessible and is available from the corresponding author upon request.

## Supplementary Materials

The following supporting information can be downloaded at: https://www.mdpi.com/article/10.3390/pathogens12111307/s1, Figure S1: The geographic distribution of the 47 Japanese prefectures and their locations; Figure S2: Probability distribution of the daily time-varying effective reproductive numbers in Japan across all the included prefectures and days; Figure S3: Temporal mean trends of the number of daily newly confirmed COVID-19 cases, effective reproduction numbers, meteorological variables, mobility patterns, and OxCGRT across all 47 Japanese prefectures and days; Figure S4: Lag–response relationship for hydrometeorological hazard scenarios; Figure S5: Lag–response relationship for hydrometeorological hazard scenarios; Figure S6: Cumulative exposure–response association for the mean ambient temperature and absolute humidity; Figure S7: Cumulative exposure–response association for the mean ambient temperature and absolute humidity; Figure S8: Cumulative exposure–response association for the mean ambient temperature and absolute humidity; Figure S9: Cumulative exposure–response association for the mean ambient temperature and absolute humidity; Figure S10: Overall exposure–response relationship between the mean ambient temperature and time-varying transmissibility of SARS-CoV-2 stratified by different levels of absolute humidity; Figure S11: Overall exposure–response relationship between the mean ambient temperature and the time-varying transmissibility of SARS-CoV-2 stratified by different levels of absolute humidity; Figure S12: Overall exposure–response relationship between the mean ambient temperature and the time-varying transmissibility of SARS-CoV-2 stratified by different levels of absolute humidity; Figure S13: Overall exposure–response relationship between the mean ambient temperature and the time-varying transmissibility of SARS-CoV-2 stratified by different levels of absolute humidity; Figure S14: Model predictions of the time-varying effective reproduction numbers in Japan; Table S1: Pairwise Spearman’s rank-order linear correlation matrix-based assessments of multicollinearity among daily meteorological variables, mobility patterns, and vaccinations across all 47 Japanese prefectures and days; Table S2: Estimated relative risks of the overall exposure–response relationship between the mean ambient temperature and the time-varying transmissibility of SARS-CoV-2 by different levels of absolute humidity over a lag of 14 days.
