# Supplementary material for: Association of Ambient Temperature and Absolute Humidity with the Effective Reproduction Number of COVID-19 in Japan
Source: Pathogens. 2023 Nov 1;12(11):1307. doi: 10.3390/pathogens12111307 (PMC10675148; doi:10.3390/pathogens12111307)
Supplement: Supplementary file 1 [file pathogens-12-01307-s001.zip › pathogens-2666527-supplementary.pdf]

Supplementary Materials for: Association of Ambient Temperature and Absolute Humidity with the Effective Reproduction Number of COVID-19 in Japan

Authors:

Keita Wagatsuma[1,2]

Affiliations:

[1] Division of International Health (Public Health), Graduate School of Medical and Dental Sciences, Niigata University, Niigata, Japan

[2] Japan Society for the Promotion of Science, Tokyo, Japan

Corresponding author:

Keita Wagatsuma

Division of International Health (Public Health)

Graduate School of Medical and Dental Sciences, Niigata University

1-757 Asahimachi-dori, Chuo-ku, Niigata City, Niigata 951-8510, Japan

Tel: +81-25-227-2129; Fax +81-25-227-0765

E-mail: waga@med.niigata-u.ac.jp

Supplementary figure legends

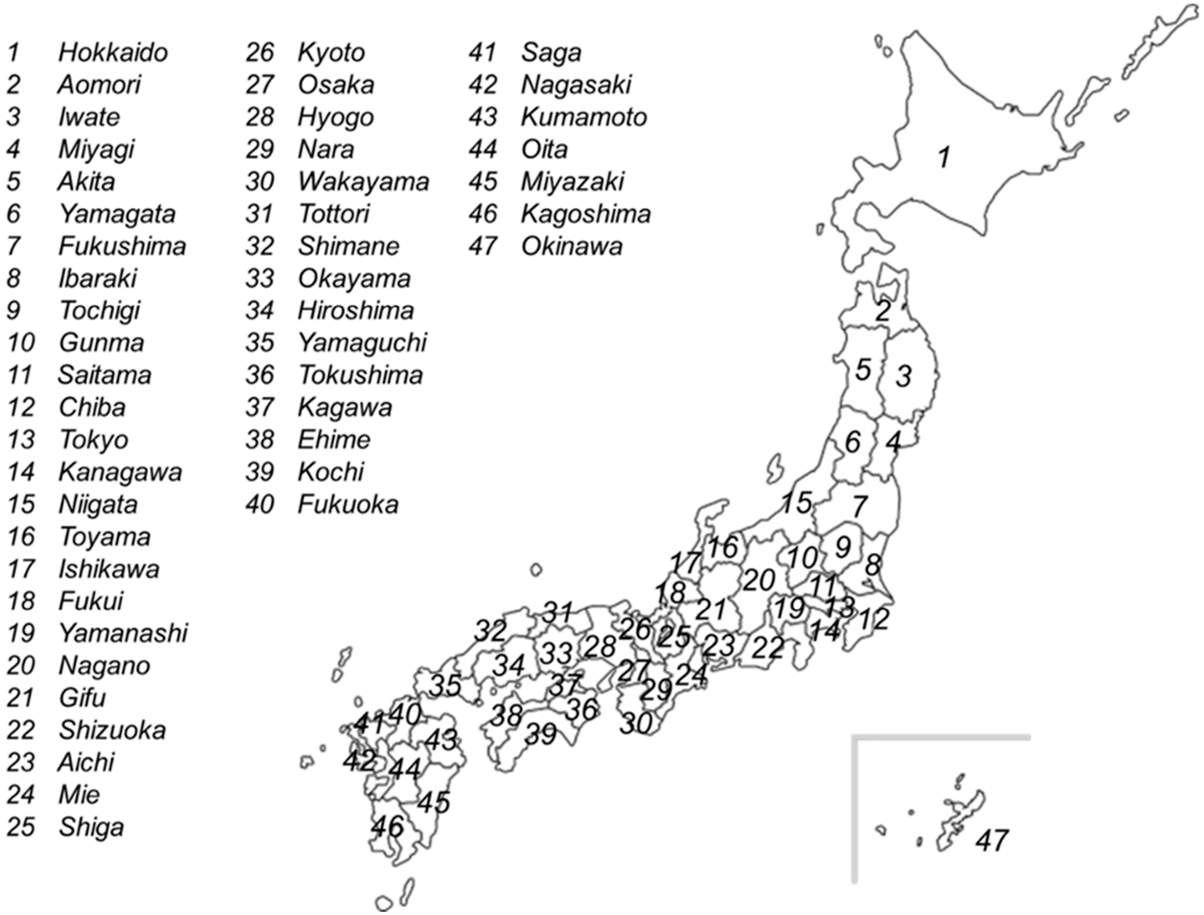

Figure S1. The geographic distribution of the 47 Japanese prefectures and their locations. Japan is located on latitudes and longitudes from approximately 26°N to 43°N and 127°E to 141°E, respectively, in the Western Pacific Region, and comprises a total of 47 prefectures, listed

from north to south as follows: Hokkaido, Aomori, Iwate, Miyagi, Akita, Yamagata, Fukushima, Ibaraki, Tochigi, Gunma, Saitama, Chiba, Tokyo, Kanagawa, Niigata, Toyama, Ishikawa, Fukui, Yamanashi, Nagano, Gifu, Shizuoka, Aichi, Mie, Shiga, Kyoto, Osaka, Hyogo, Nara, Wakayama, Tottori, Shimane, Okayama, Hiroshima, Yamaguchi, Tokushima, Kagawa, Ehime, Kochi, Fukuoka, Saga, Nagasaki, Kumamoto, Oita, Miyazaki, Kagoshima, and Okinawa.

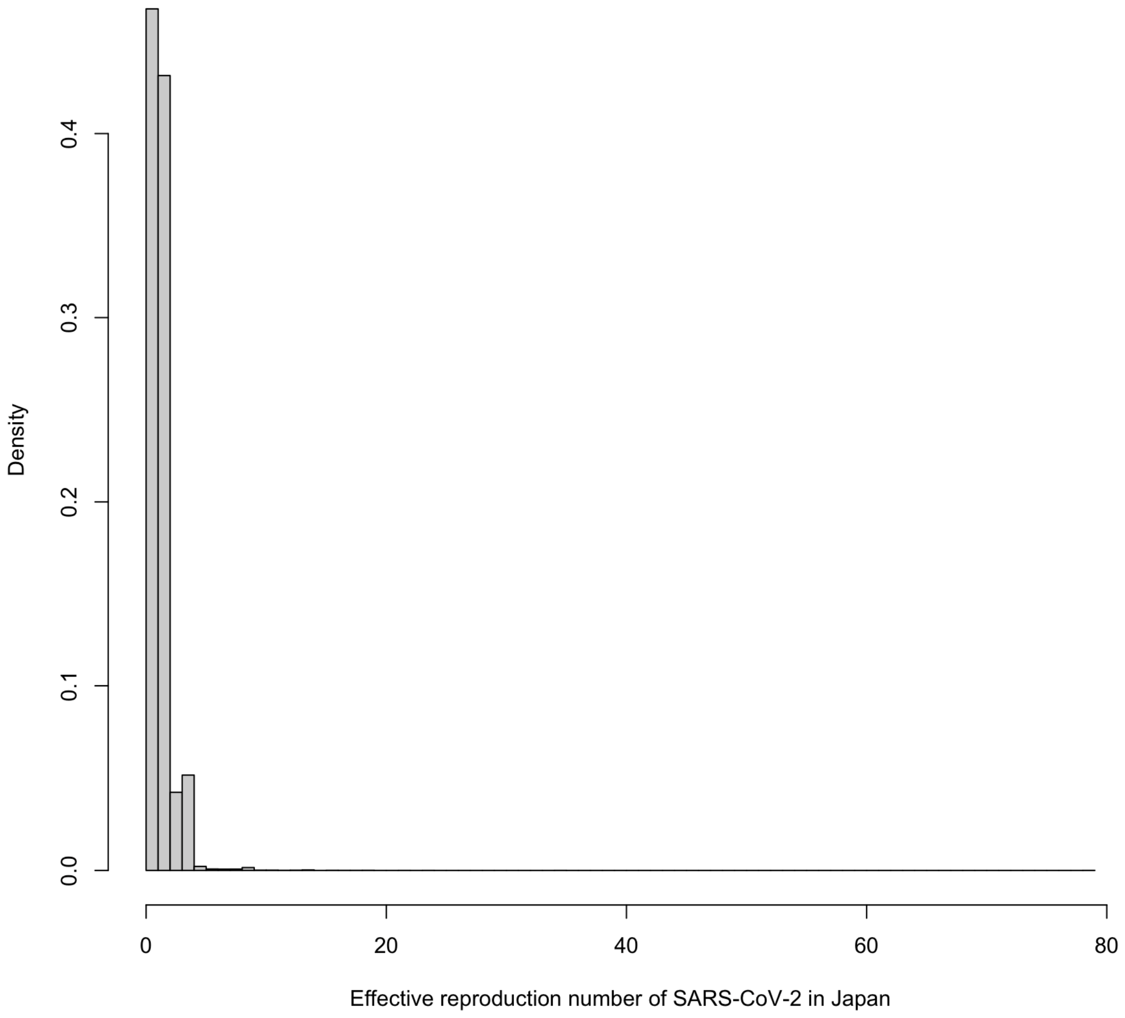

Density
0.0
0.1
0.2
0.3
0.4
0
20
40
60
80
Effective reproduction number of SARS-CoV-2 in Japan

Figure S2. Probability distribution of the daily time-varying effective reproductive numbers in Japan across all the included prefectures and days. The mean daily time-varying effective reproduction number in Japan across all the included prefectures and days was 1.24. These observational data do not follow a normal distribution (Shapiro–Wilk test, $P < 0.001$).

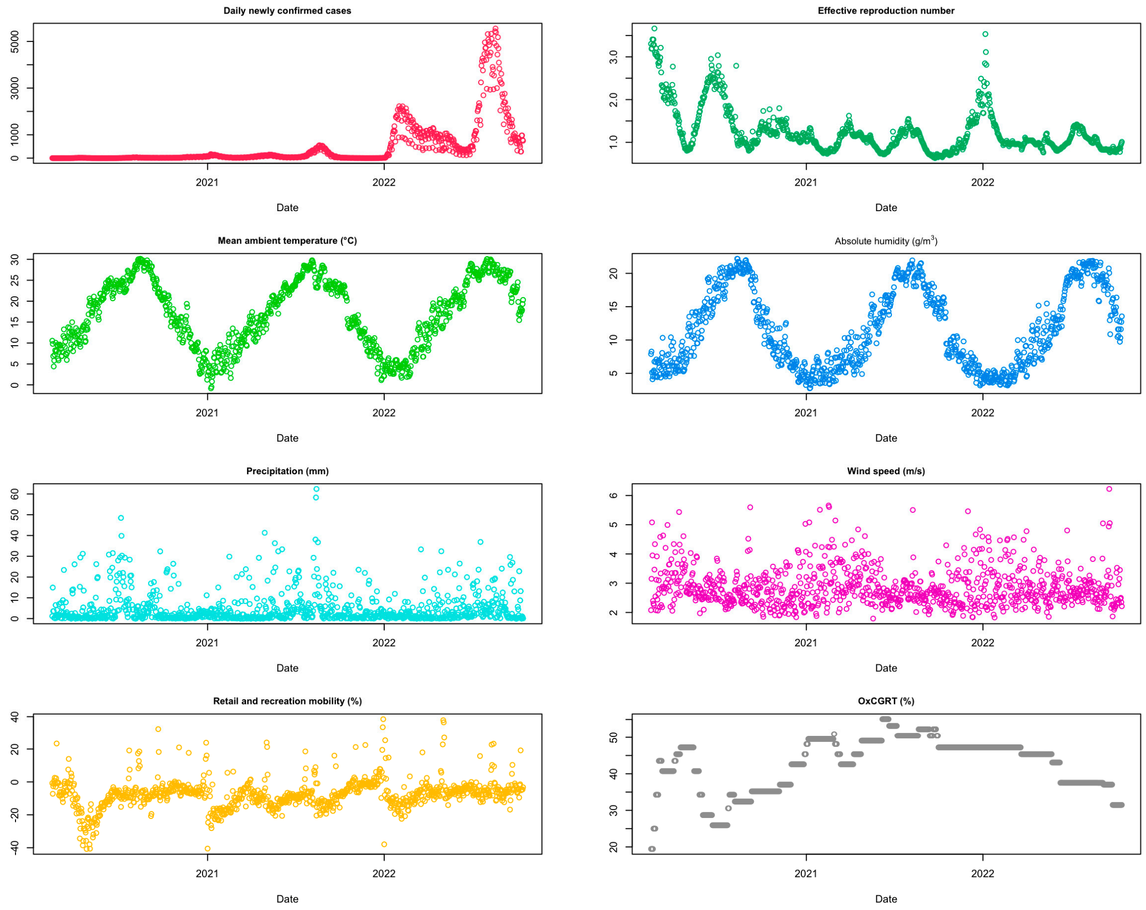

Figure S3. Temporal mean trends of the number of daily newly confirmed COVID-19 cases, effective reproduction numbers, meteorological variables, mobility patterns, and OxCGRT across all 47 Japanese prefectures and days. Abbreviations: OxCGRT: Oxford Coronavirus Government Response Tracer.

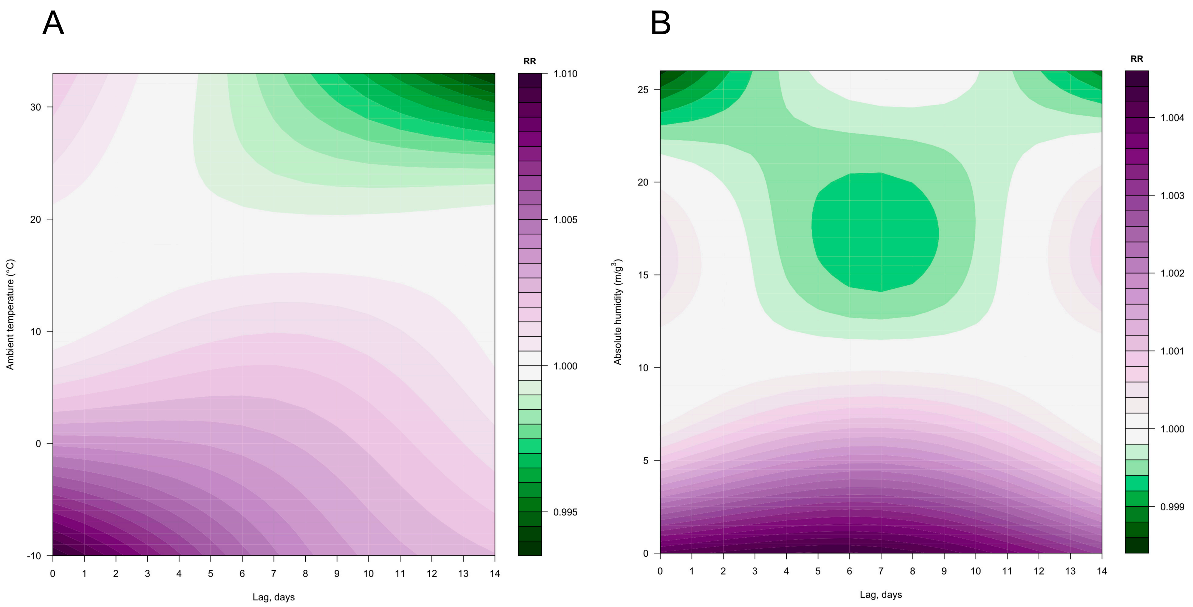

Figure S4. Lag–response relationship for hydrometeorological hazard scenarios. Contour plot of the association between the (**A**) daily mean ambient temperature (°C) and risk of estimated time-varying effective reproduction number (R_t) over a 14-day lag, relative to the overall median of the mean ambient temperature (17.8°C), and (**B**) daily absolute humidity (m/g^3) and risk of estimated time-varying R_t over a 14-day lag, relative to the overall median of absolute humidity (10.6 m/g^3). The deeper the shade of purple, the greater the increase in the relative risks (RR) of the estimated time-varying R_t as compared with the overall mean ambient temperature. The deeper the shade of green, the greater the decrease in the RR of estimated time-varying R_t as compared with the overall mean ambient temperature.

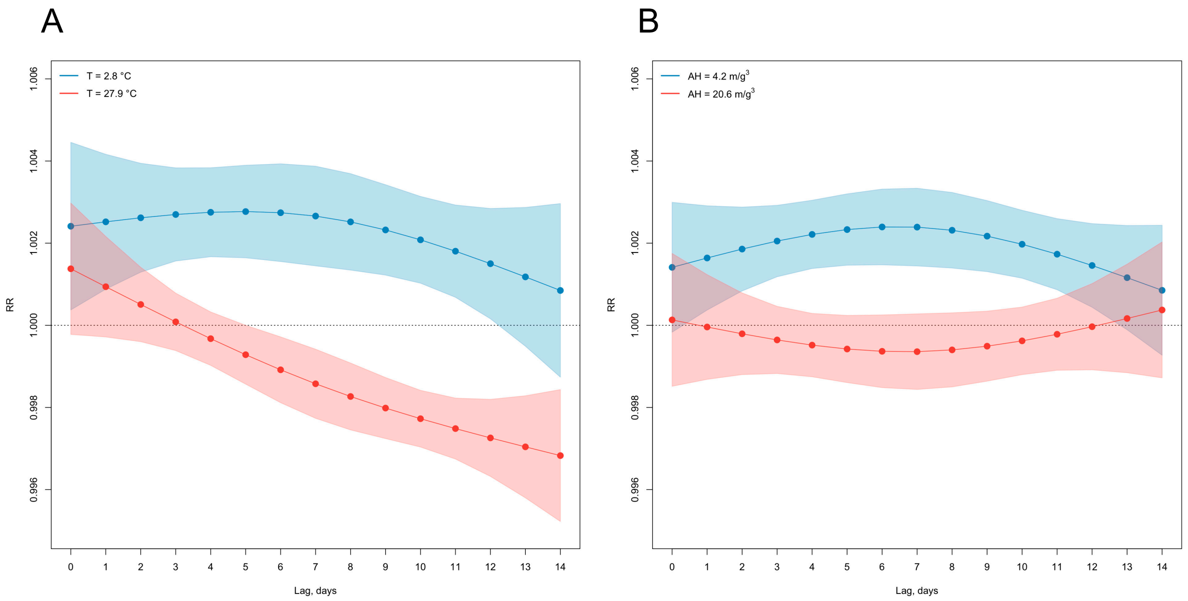

Figure S5. Lag–response relationship for hydrometeorological hazard scenarios. Lag–response association for scenarios of (**A**) mean ambient temperature: exceptionally cold (5.1°C; 10th percentile) and exceptionally hot (27.9°C; 90th percentile) conditions relative to the baseline (17.8°C; 50th percentile) and (**B**) absolute humidity: 4.2 m/g^3 (10th percentile) and 20.6 m/g^3 (90th percentile) relative to the baseline (10.6 m/g^3; 50th percentile), at lags between 0 and 14 days. the shaded areas represent 95% confidence intervals (CI). Abbreviations: AH, absolute humidity; RR, relative risk; T, mean ambient temperature.

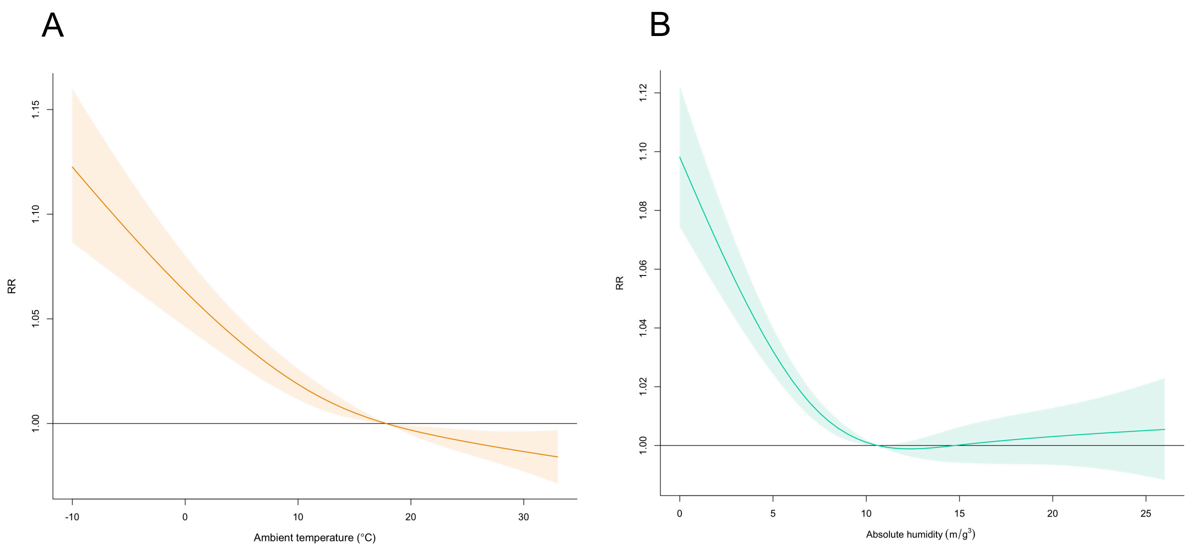

Figure S6. Cumulative exposure–response association for the mean ambient temperature and absolute humidity. Overall exposure–response association across all lags (0–14 days) between the time-varying effective reproduction number (R_t) of SARS-CoV-2 and (**A**) the mean ambient temperature (°C) relative to the overall mean minimum ambient temperature of 17.8°C and (**B**) absolute humidity (m/g^3) relative to the overall mean maximum ambient temperature of 10.6 m/g^3. The shaded areas represent the 95% confidence intervals. In this sensitivity analysis, a natural cubic spline of time was set up with different degrees of freedom (four degrees of freedom per year).

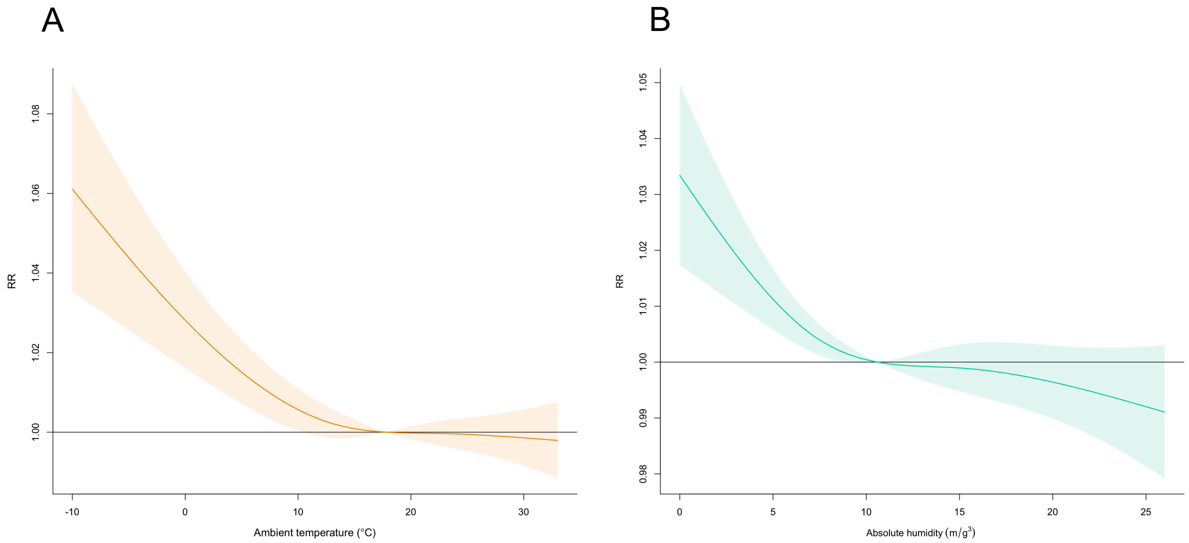

Figure S7. Cumulative exposure–response association for the mean ambient temperature and absolute humidity. The overall exposure–response association across all lags (0–7 days) between the time-varying effective reproduction number (R_t) of SARS-CoV-2 and the (**A**) mean ambient temperature (°C) relative to the overall mean minimum ambient temperature of 17.8°C and (**B**) absolute humidity (m/g^3) relative to the overall mean maximum temperature of 10.6 m/g^3. The shaded areas represent the 95% confidence intervals. In this sensitivity analysis, the lagged structure of the cross-basis function was changed from 0–14 to 0–7 days.

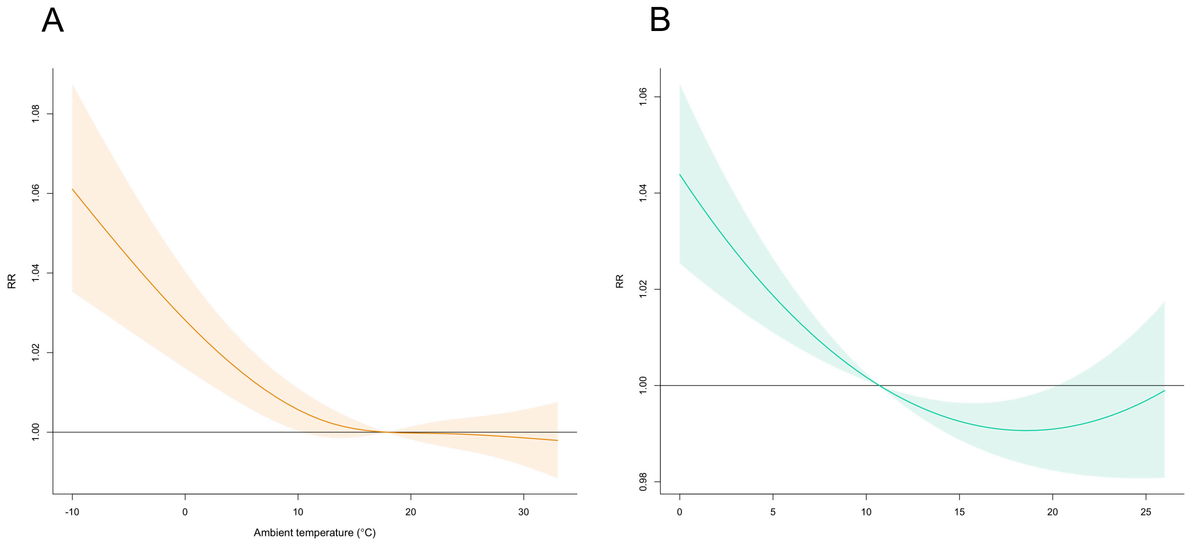

Figure S8. Cumulative exposure–response association for the mean ambient temperature and absolute humidity. The overall exposure–response association across all lags (0–14 days) between the time-varying effective reproduction number (R_t) of SARS-CoV-2 and (**A**) the mean ambient temperature (°C) relative to the overall mean minimum temperature of 17.8°C and (**B**) absolute humidity (m/g^3) relative to the overall mean maximum ambient temperature of 10.6 m/g^3. The shaded areas represent the 95% confidence intervals. In this sensitivity analysis, the functional form of spline for the exposure–response curve ranges from a natural cubic spline with 3 degrees of freedom (df) to a second-degree polynomial. The lag–response curve was remodeled with a natural cubic spline with intercept and two equally spaced knots on a log scale.

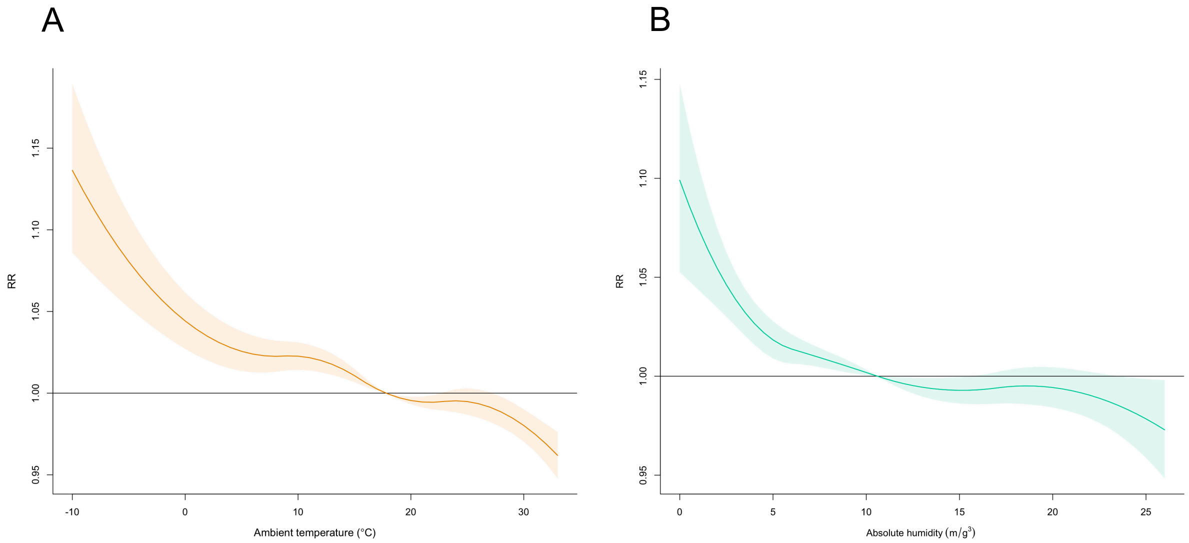

Figure S9. Cumulative exposure–response association for the mean ambient temperature and absolute humidity. The overall exposure–response association across all lags (0–14 days) between the time-varying effective reproduction number (R_t) of SARS-CoV-2 and (**A**) the mean ambient temperature (°C) relative to the overall mean minimum ambient temperature of 17.8°C and (**B**) absolute humidity (m/g^3) relative to the overall mean maximum ambient temperature of 10.6 m/g^3. The shaded areas represent the 95% confidence intervals. In this sensitivity analysis, the functional form of spline for the exposure–response curve extends from a natural cubic spline with 3 degrees of freedom (df) to a quadratic B-spline with three internal knots, placed at the 25th, 50th, and 75th percentiles of the mean ambient temperature and absolute humidity. The lag–response curve was remodeled with a natural cubic spline with intercept and two equally spaced knots on a log scale.

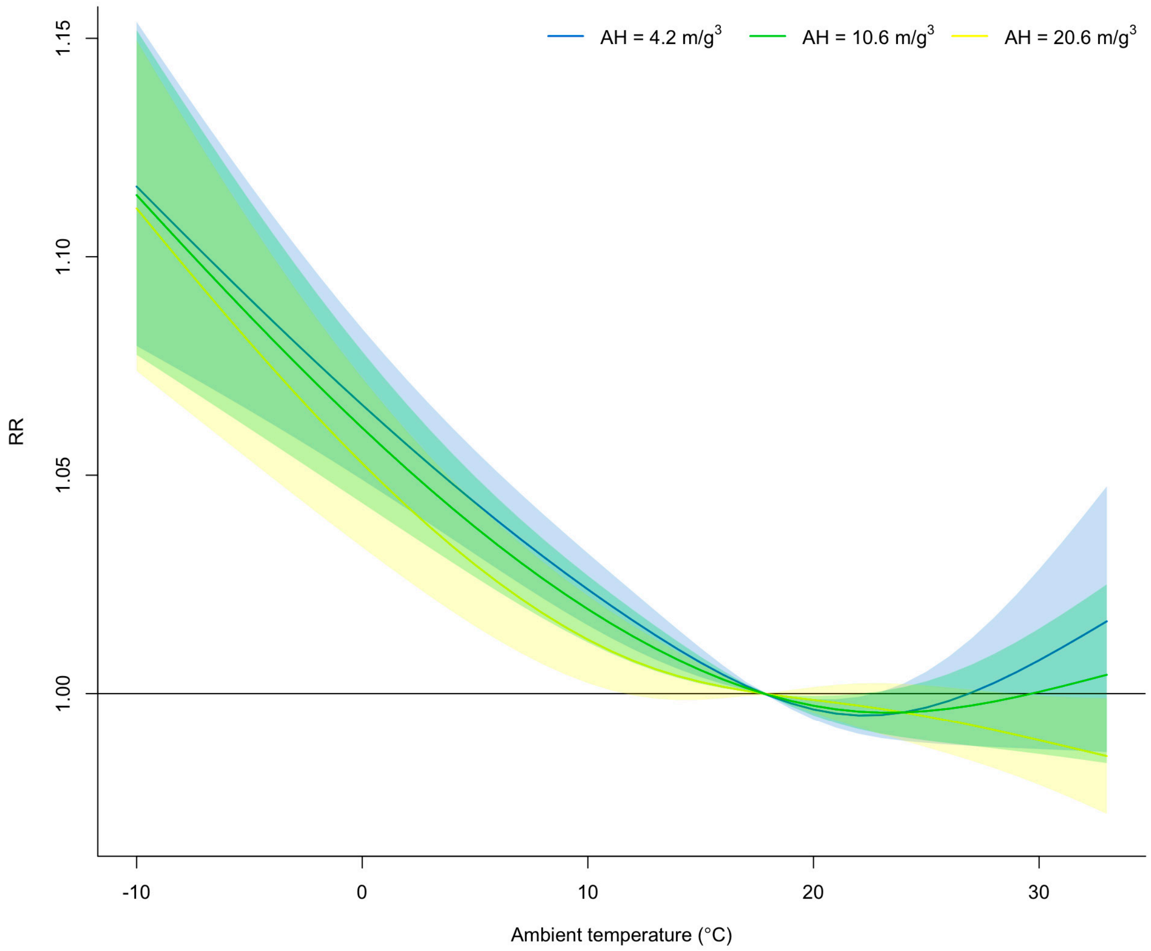

AH = 4.2 m/g^3
AH = 10.6 m/g^3
AH = 20.6 m/g^3
RR
1.15
1.10
1.05
1.00
-10
0
10
20
30
Ambient temperature (°C)

Figure S10. Overall exposure–response relationship between the mean ambient temperature and time-varying transmissibility of SARS-CoV-2 stratified by different levels of absolute humidity. The pooled relative risk (RR) was centered at the overall median of mean ambient temperature (17.8°C; 50th percentile); high absolute humidity (AH) group (20.6 m/g^3; 90th percentile;), medium absolute humidity group (10.6 m/g^3; 50th percentile), and low absolute humidity group (4.2 m/g^3; 10th percentile). The shaded areas on the curves are the 95% confidence interval. In this sensitivity analysis, a natural cubic spline of time was set up with different degrees of freedom (four degrees of freedom per year).

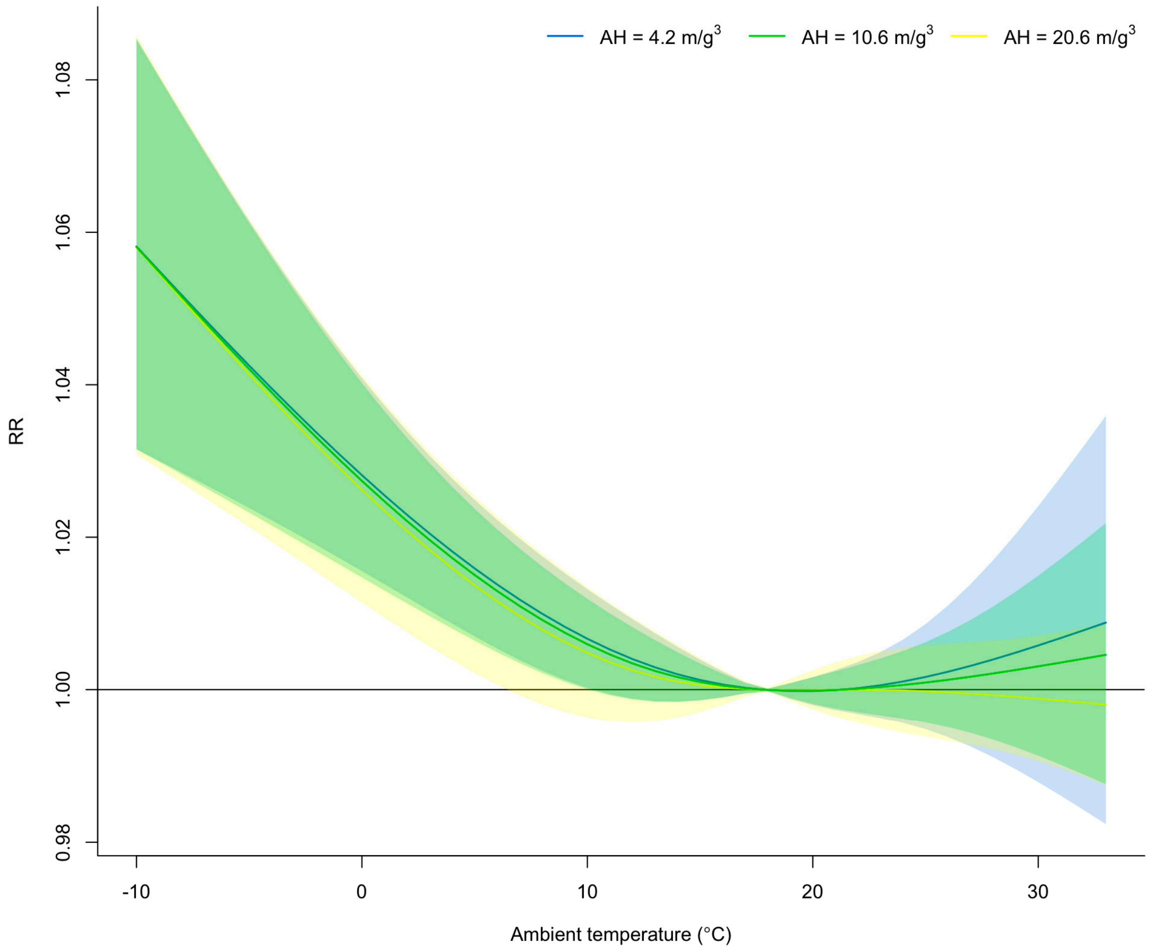

Figure S11. Overall exposure–response relationship between the mean ambient temperature and the time-varying transmissibility of SARS-CoV-2 stratified by different levels of absolute humidity. The pooled relative risk (RR) was centered at the overall median of mean ambient

temperature (17.8°C; 50th percentile); high absolute humidity (AH) group (20.6 m/g^3; 90th percentile;), medium absolute humidity group (10.6 m/g^3; 50th percentile), and low absolute humidity group (4.2 m/g^3; 10th percentile). The shaded areas on the curves are the 95% confidence interval. In this sensitivity analysis, the lagged structure of the cross-basis function was changed from 0–14 to 0–7 days.

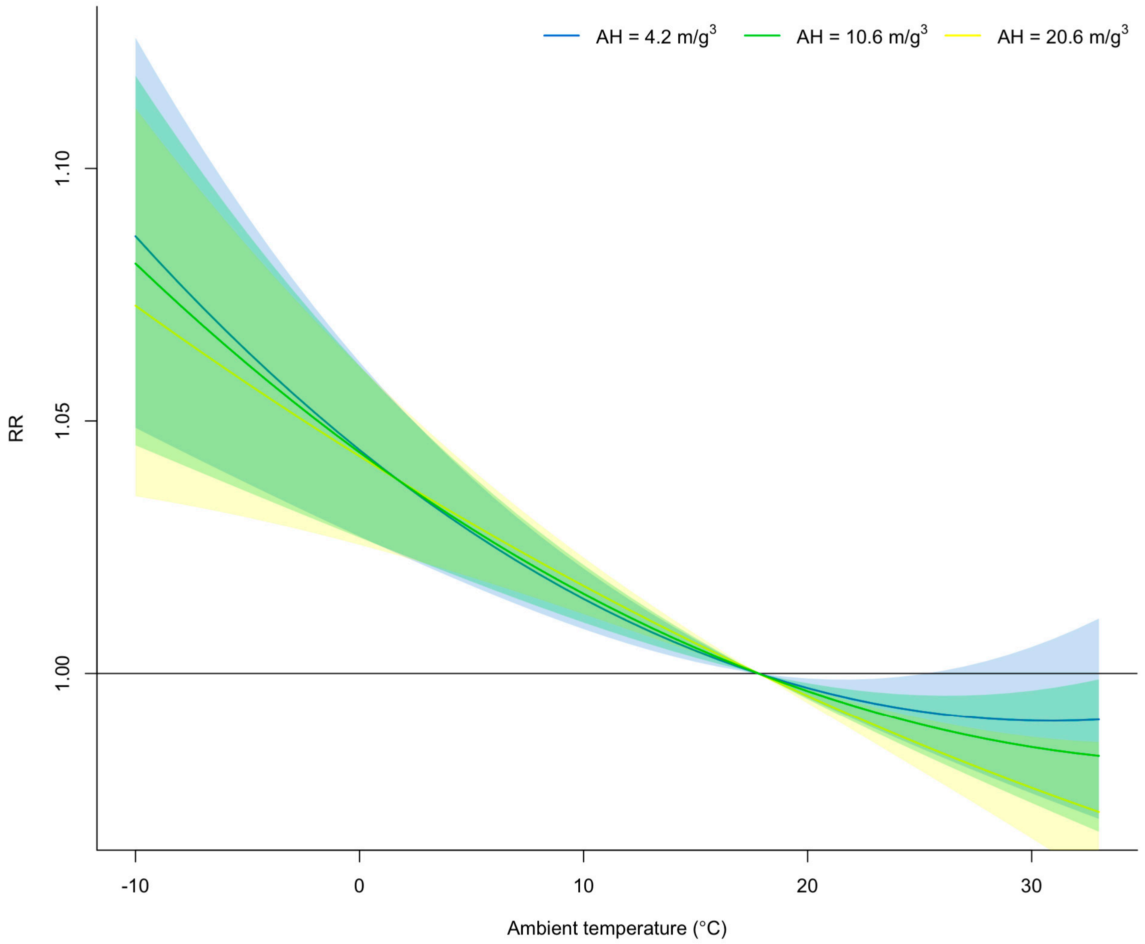

Figure S12. Overall exposure–response relationship between the mean ambient temperature and the time-varying transmissibility of SARS-CoV-2 stratified by different levels of absolute humidity. The pooled relative risk (RR) was centered at the overall median of mean ambient

temperature (17.8°C; 50th percentile); high absolute humidity (AH) group (20.6 m/g^3; 90th percentile;), medium absolute humidity group (10.6 m/g^3; 50th percentile), and low absolute humidity group (4.2 m/g^3; 10th percentile). The shaded areas on the curves are the 95% confidence intervals. In this sensitivity analysis, the functional form of the spline for the exposure–response curve extended from a natural cubic spline with 3 degrees of freedom (df) to a second-degree polynomial. The lag–response curve was remodeled with a natural cubic spline with intercept and two equally spaced knots on a log scale.

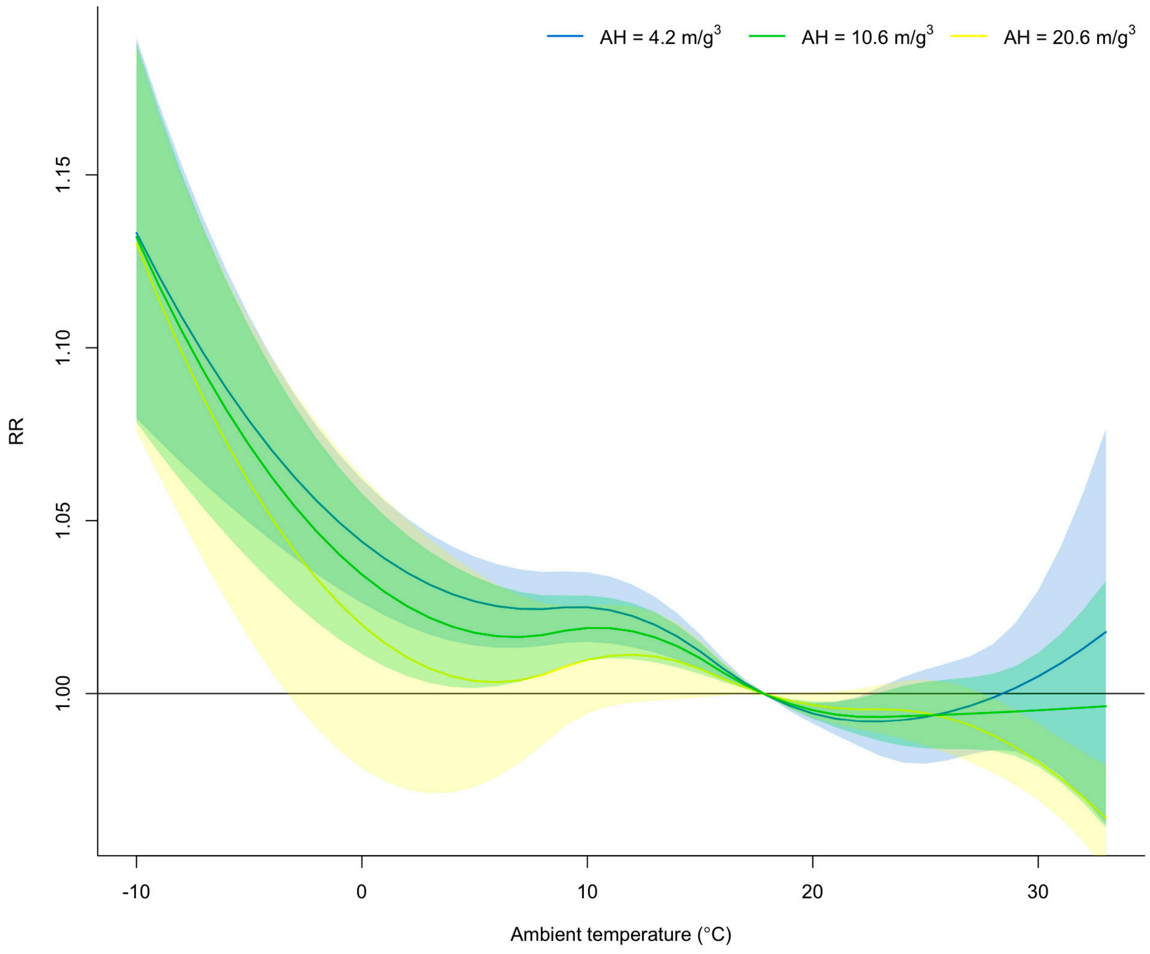

AH = 4.2 m/g3
AH = 10.6 m/g3
AH = 20.6 m/g3
RR
1.15
1.10
1.05
1.00
-10
0
10
20
30
Ambient temperature (°C)

Figure S13. Overall exposure–response relationship between the mean ambient temperature and the time-varying transmissibility of SARS-CoV-2 stratified by different levels of absolute humidity. The pooled relative risk (RR) was centered at the overall median of the mean ambient temperature (17.8°C; 50th percentile) with the high absolute humidity (AH) group (20.6 m/g^3; 90th percentile), medium absolute humidity group (10.6 m/g^3; 50th percentile), and low absolute humidity group (4.2 m/g^3; 10th percentile). The shaded areas on the curves are the 95% confidence interval. In this sensitivity analysis, the functional form of spline for the exposure–response curve varied from a natural cubic spline with 3 degrees of freedom (df) to a quadratic B-spline with three internal knots, placed at the 25th, 50th, and 75th percentiles of the mean ambient temperature and absolute humidity. The lag–response curve was remodeled with a natural cubic spline with intercept and two equally spaced knots on a log scale.

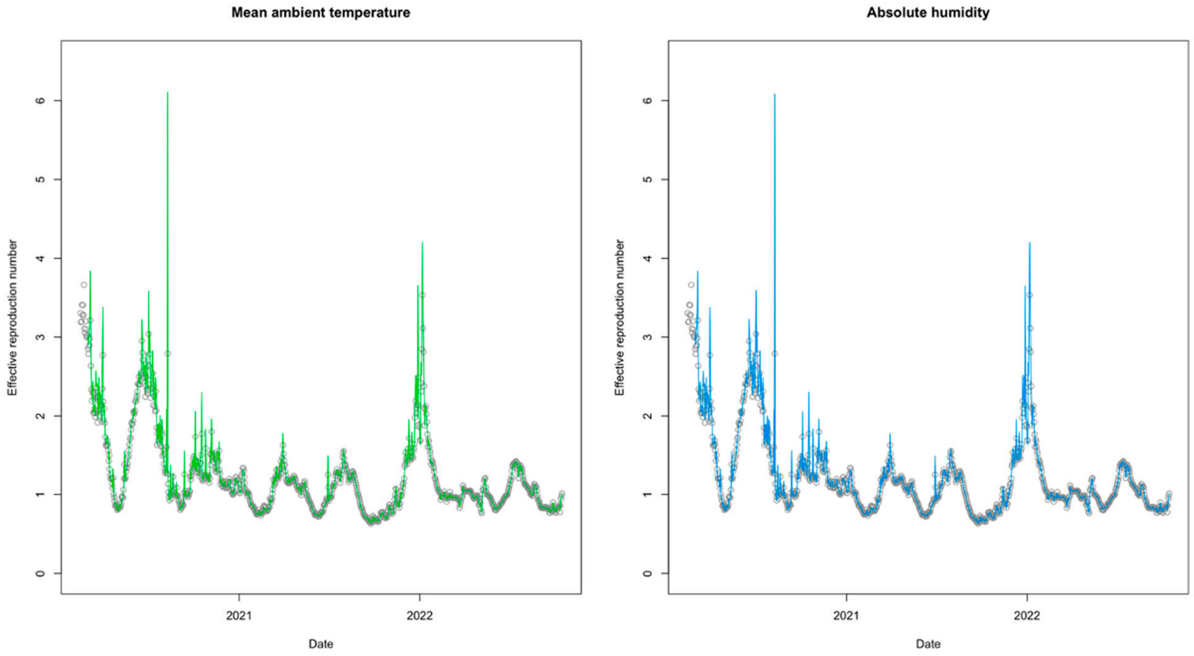

Figure S14. Model predictions of the time-varying effective reproduction numbers in Japan. The gray dots represent the observed temporal mean trends of the time-varying effective reproduction numbers (R_t) of SARS-CoV-2 across all 47 Japanese prefectures. The green and blue solid lines represent the time-varying R_t predicted (in-sample) from the main model including the cross-basis functions for (**A**) mean ambient temperature and (**B**) absolute humidity, respectively. Pair-wise Spearman's rank-order linear correlation coefficients statistics showed that the temporal mean trends of the time-varying R_t strongly and positively correlated with time-varying R_t predicted from the main model of mean ambient temperature (ρ = 0.99) and absolute humidity (ρ = 0.99), respectively.

Table S1. Pairwise Spearman's rank-order linear correlation matrix-based assessments of multicollinearity among daily meteorological variables, mobility patterns, and vaccinations across all 47 Japanese prefectures and days.

Potential drivers	1	2	3	4	5	6	7
1. Effective reproductive number	1.00						
2. Mean ambient temperature (℃)	−0.08***	1.00					
3. Absolute humidity (m/g³)	−0.06***	0.95***	1.00				
4. Precipitation (mm)	0.03***	0.04***	0.22***	1.00			
5. Wind speed (m/s)	0.01	−0.04***	−0.10***	0.03***	1.00		
6. Retail and recreation mobility (%)	0.11***	−0.01**	−0.01***	−0.13***	−0.07***	1.00	
7. OxCGRT (%)	−0.23***	−0.22***	−0.23***	−0.02***	0.03***	−0.23***	1.00

Abbreviations: OxCGRT: Oxford Coronavirus Government Response Tracer. Notes: Significant predictors in the statistical model are described by $^{*}P < 0.05$; $^{**}P < 0.01$; $^{***}P < 0.001$.

Table S2. Estimated relative risks of the overall exposure–response relationship between the mean ambient temperature and the time-varying transmissibility of SARS-CoV-2 by different levels of absolute humidity over a lag of 14 days.

**Subgroups**	**Low mean ambient temperature (°C), RR (95% CI)**		**High mean ambient temperature (°C), RR (95% CI)**	
	**2.8°C (5th percentile)**	**5.1°C (10th percentile)**	**27.9°C (90th percentile)**	**29.9°C (95th percentile)**
Low AH	1.039 (1.025–1.053)	1.032 (1.020–1.044)	0.995 (0.981–1.010)	0.997 (0.977–1.017)
Medium AH	1.034 (1.020–1.048)	1.028 (1.016–1.039)	0.990 (0.980–1.001)	0.990 (0.976–1.004)
High AH	1.027 (1.011–1.044)	1.021 (1.007–1.035)	0.983 (0.975–0.992)	0.979 (0.969–0.989)

Abbreviations: AH, absolute humidity; CI, confidence interval; RR, relative risk; SARS-CoV-2, severe acute respiratory syndrome coronavirus 2. Notes: 2.8°C, 5.1°C, 27.9°C, and 29.9°C correspond to the 5th, 10th, 90th, and 95th percentiles of mean ambient temperature, respectively. The pooled RR was centered at the overall median of the mean ambient temperature (17.8°C; 50th percentile); high AH group: 20.6 m/g^3 (90th percentile); medium AH group: 10.6 m/g^3 (50th percentile); and low AH group: 4.2 m/g^3 (10th percentile).
